# Over 1.65 GW cm^−2^ sr^−1^ brightness 590 nm yellow second-harmonic generation in MOCVD-grown high-strain InGaAs/GaAs quantum well VECSEL

**DOI:** 10.1038/s41377-026-02230-8

**Published:** 2026-03-10

**Authors:** Zhicheng Zhang, Wenbo Zhan, Yao Xiao, Chen Luo, Hao Zhou, Wenfan Yang, Yang Cheng, Hao Yu, Quanling Li, Xiao Li, Chaofan Zhang, Jun Wang

**Affiliations:** 1 College of Advanced Interdisciplinary Studies, National University of Defense Technology, Changsha, 410073 China; 2 Suzhou Everbright Photonics Co., Ltd, Suzhou, 215163 China; 3 College of Electronics and Information Engineering, Sichuan University, Chengdu, 610064 China

**Keywords:** Semiconductor lasers, Solid-state lasers

## Abstract

High-brightness yellow lasers are in high demand for applications such as atomic cooling and trapping, optogenetics, and sodium laser guide stars. Herein, we demonstrate the potential of Metal-Organic Chemical Vapor Deposition (MOCVD) for the rapid mass production of high-strain 1.2 μm InGaAs quantum well vertical external cavity surface emitting lasers (VECSELs). Two distinct growth strategies were explored, with a primary focus on enhancing crystal thermal stability and mitigating indium segregation. The as-grown gain chips achieved over 45 W of output power and a slope efficiency exceeding 50%. Furthermore, we verified the feasibility of generating yellow second harmonic generation (SHG), attaining a 590 nm CW power of ~6.2 W with a slope efficiency of 17%. The beam quality factor (*M*²) was <1.1, approaching diffraction-limited performance, corresponding to a brightness of ~1.65 GW cm^−2^ sr^−1^. Overall, these investigations not only expand the performance envelope of MOCVD-grown semiconductor lasers but also deepen the understanding of indium segregation behaviors.

## Introduction

Yellow-laser sources have emerged as highly desirable tools across diverse fields, including atomic cooling and trapping^1^, optogenetics^2,3^, ophthalmic diagnosis/treatment^4,5^, and sodium laser guide stars^6,7^. Hemoglobin and oxyhemoglobin strongly absorb yellow light; consequently, yellow laser-based phototherapy has been employed to treat corneal diseases, aortic tumors, and skin conditions^8,9^. Additionally, 589 nm lasers offer a compelling application: their ability to excite the *D₂* spectral line, resonantly interacting with the sodium atom layer at altitudes of 80–100 km to generate high-brightness fluorescent radiation in the reverse direction^10^. The sodium laser guide star plays a vital role in adaptive optics imaging correction systems, significantly enhancing the imaging resolution of astronomical telescopes. Similarly, related technology is applied in sodium Doppler lidar, enabling measurements of temperature and wind fields in the middle and upper atmosphere^11^. These intriguing applications have sparked a growing interest in yellow lasers, and the development of high-power, high-beam-quality yellow lasers has been a hot topic over the past three decades^9^.

**Fig. 1:**
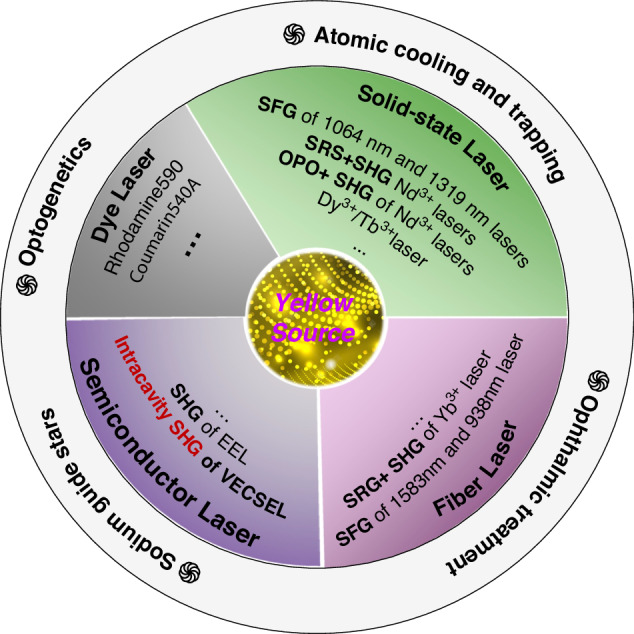
Various laser technologies for attaining yellow emission. SHG Second Harmonic Generation, SFG Sum Frequency Generation, SRS Stimulated Raman Scattering, OPO Optical Parametric Oscillator, EEL Edge-emitting laser

As depicted in Fig. 1, the approaches to obtaining yellow lasers can be categorized into dye, solid-state, fiber, and semiconductor lasers. Initially, yellow lasers were directly generated by dye lasers. Nevertheless, on account of its enormous size and its well-known unreliability issues, it ultimately fell into disuse^6^. The second technology is the solid-state and fiber laser, which has undergone rapid advancement in the 21st century. However, due to constraints imposed by energy level structures, directly generating high-quality gain within the yellow even or 1.2 μm band remains challenging. Consequently, a prevalent strategy involves first generating 1.2 μm lasers via diverse nonlinear effects (such as stimulated Raman scattering (SRS)^12^, and optical parametric oscillation (OPO)^13,14^), followed by frequency doubling to produce a yellow laser. Although these approaches have exhibited considerable potential in achieving high power and superior beam quality, they are still plagued by limitations, including large laser system volumes and low wall-plug efficiency. Alternatively, newly developed Dy³⁺/Tb³⁺-doped lasers and vertical external-cavity surface-emitting lasers (VECSELs) represent a third category of solutions for generating yellow lasers. Dy³⁺/Tb³⁺-doped lasers are capable of directly producing yellow emission; however, achieving high power remains an unmet challenge^15,16^. By contrast, VECSELs, featuring the tunable bandgap design of semiconductors, can achieve high-performance 1.2 μm emission. The external cavity further facilitates second harmonic generation (SHG) within the resonator, thereby yielding higher SHG efficiency^17^.

Overall, the VECSEL is a promising solution for high-brightness yellow laser sources, but mitigating the excessive accumulated compressive strain with increasing indium composition remains a major challenge^18–22^. For the InGaAs/GaAs material system, when the quantum well achieves gain at approximately 1.2 μm, the indium content reaches around 40%, and the lattice mismatch between InGaAs and GaAs substrates is roughly 3%, leading to the formation of misfit dislocations. To overcome strain limitations, several instructive methods have been employed, such as InGaNAs/GaAs QWs (11 W)^23^, InGaAs/GaAs quantum dots (2 W)^24^, and GaAsSb/GaAs QWs (4 W)^25,26^. Alternatively, to alleviate this issue, another promising solution involves employing strain compensation technology and optimizing epitaxial growth parameters. Molecular Beam Epitaxy (MBE) operates under ultra-high vacuum, with instant beam flux regulation and low growth temperature enabling atomic-level precision, abrupt interfaces, and strain tuning. The output power of 1.2 μm MBE-grown VECSELs has steadily improved: 7 W^27^, 20 W^28^, 50 W^29^, and recently 72 W^30^. In contrast, Metal-Organic Chemical Vapor Deposition (MOCVD) offers faster growth and better mass production, but 1.2 μm VECSELs’ performance remains below 10 W^31^—far inferior to MBE. This is presumably because epitaxial growth based on faster gaseous reactants, coupled with higher growth temperatures, presents greater challenges for strain and interface control. As a result, large-scale and commercial fabrication of 1.2 μm VECSEL chips remains challenging.

In this paper, we demonstrate the potential of MOCVD for the rapid mass production of high-strain 1.2 μm VECSELs. Two distinct growth strategies are explored, with a focus on investigating improvements in crystal thermal stability and mitigating the indium segregation phenomenon. The as-grown gain chips achieve over 45 W of output power and a slope efficiency exceeding 50% under continuous-wave operation. Furthermore, we verify the feasibility of generating 590 nm yellow second harmonic generation, attaining an SHG power of ~6.2 W and a slope efficiency of 17%. The beam quality factor (*M*²) is <1.1, approaching diffraction-limited performance, corresponding to a calculated brightness of ~1.65 GW cm^−2^ sr^−1^. Overall, these studies substantially expand the performance envelope of MOCVD-grown semiconductor lasers, thus extending and advancing prior work in this field.

## Results

### Structure and strain optimization

**Fig. 2:**
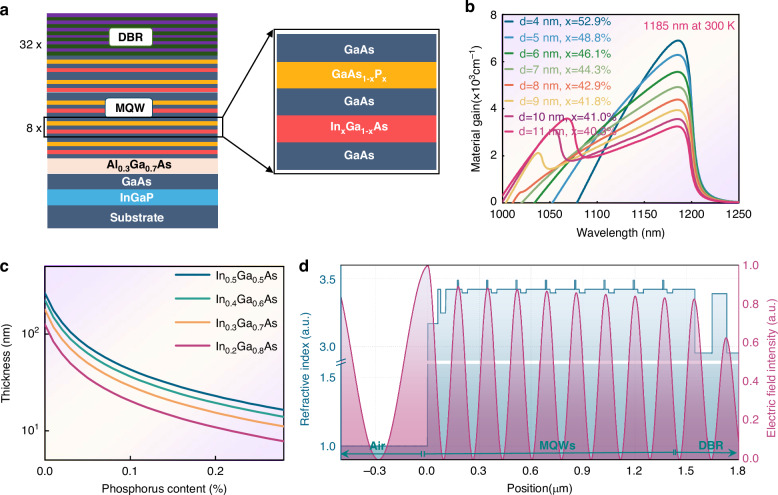
Strain and Structure Optimization. **a** The designed “flip-chip” epitaxial structure; **b** Calculated gain spectra of the QWs with different thicknesses and indium contents; **c** Calculated GaAsP thickness required to fully compensate the InGaAs layer; **d** Refractive index and standing-wave electric field distribution within the chip

The epitaxial structure is designed in a “flip-chip” configuration, as depicted in Fig. 2a. On the GaAs substrate, InGaP and Al₀.₃Ga₀.₇As are incorporated, functioning as the etch stop layer and window layer, respectively. Subsequently, eight-period InGaAs/GaAs quantum wells (QWs) are employed to generate optical gain. Employing the Crosslight™ PIC3D software, the QWs’ gain is evaluated under a temperature of 300 K and a carrier density of 5 × 10^18 ^cm^−3^. As can be observed from Fig. 2b, when the gain peak is at 1185 nm, the indium content exceeds 40%, corresponding to a lattice mismatch with the GaAs substrate of about 3%. To maintain the peak, narrowing the QW thickness requires increasing the indium content, which may exacerbate the lattice mismatch. Beyond 8 nm, a secondary gain peak emerges due to weakened quantum confinement^32^. Thus, a wider 7 nm QW is adopted to reduce the mismatch, while also providing optimal overlap between the QWs and the standing wave electric field. Furthermore, a tensile-strained GaAsP layer is introduced to compensate for the compressive strain of InGaAs^33^. Using the zero-strain method^34^, the calculated GaAsP thickness required to fully compensate for InGaAs layers of varying compositions is mapped in Fig. 2c. On the other hand, since photo-generated carriers must reach the QW through the GaAsP layer for recombination, phosphorus composition cannot be too high—higher P increases the GaAsP bandgap, impeding transport. The calculated energy band structure is provided in Fig. S1 in the Supplemental document. Here, a 32-nm-thick GaAs₀.₉P₀.₁ layer is designed to compensate for the 7-nm-thick quantum well layer, resulting in an overall net lattice mismatch of approximately 0.15%. Moderate compressive strain enables better matching between the valence and conduction band densities of states, yielding higher differential gain and a lower transparency carrier density^32^. Another concern is that phosphorus atoms may cause arsenic-phosphorus intermixing at the interface, forming random InGaAsP quaternary compounds^35^. This leads to interface roughness, increased photon scattering, a broadened gain spectrum, and degraded optical gain^36^. To mitigate this, a GaAs insertion layer is added between the InGaAs and GaAsP layers. Finally, thirty-two pairs of Al₀.₉Ga₀.₁As/Al₀.₀₆Ga₀.₉₄As distributed Bragg reflectors (DBRs) are designed after the active region, with their high-reflectivity band centered at 1180 nm. Figure 2d shows the refractive index and standing-wave electric field distribution within the chip. The QWs are positioned at the standing-wave antinodes to form the resonant periodic gain structure^37^, thereby enhancing stimulated emission probability and improving optical gain utilization efficiency^38^.

### Epitaxial growth and characterization

**Fig. 3:**
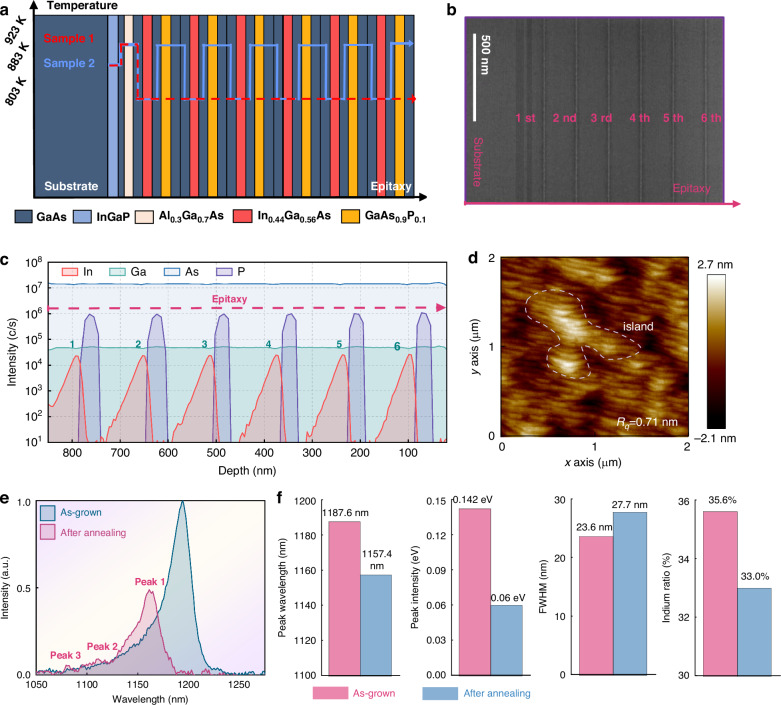
Epitaxial Growth and Characterization of Sample 1. **a** The growth temperature of each layer is shown, with the red and blue lines representing two different samples; **b** The transmission electron microscopy image; **c** Measured secondary ion mass spectrometry (SIMS); **d** Atomic force microscope; **e** Photoluminescence spectrum before and after annealing; **f** Statistical PL peak wavelength, peak intensity, full width, and indium component at a six-inch wafer

Indium segregation is an unwanted issue in the growth of high-indium-content InGaAs QW, and numerous previous studies have been dedicated to optimizing this phenomenon^39–42^. Owing to its larger atomic size, indium induces lattice distortion in the crystal, which increases surface defects and reduces atomic density, thus driving indium migration along the crystal growth direction^43^. As a result, this issue causes surface roughening and triggers a transition in growth mode from two-dimensional to three-dimensional island-like growth. The diffusion rate exhibits a marked sensitivity to temperature: $R=1/\exp (\frac{{E}_{s}}{{kT}})$, where *R* and *E*_*s*_ are the segregation coefficient and segregation energy^44^. Thus, previous explorations have shown that a low-growth temperature helps suppress the diffusion of indium atoms and ameliorates the segregation^20,21,45^. Employing this method, the designed multi-active regions are verified. The quantum well period was initially set to 6 periods, and the top Bragg reflector structure was omitted. The epitaxial layers were grown on an n-type 2 ° GaAs substrate using an MOCVD system (Aixtron™, 2800G4). Triethylgallium, trimethylindium, arsine, and phosphine were used as source materials, with hydrogen as the carrier gas. The Triethylgallium source has a low decomposition temperature, minimizing impurity introduction via *β*-hydride elimination reactions^46^. The growth temperature profile is shown as the red line in Fig. 3a: The substrate is first heated to 883 K to remove surface impurities, and the InGaP layer is grown at this temperature. The temperature is then increased to 923 K for growing the GaAs and Al₀.₃Ga₀.₇As layers. For the subsequent active region growth, the temperature is reduced to 803 K and held constant (this configuration is designated Sample 1). After growth, high-temperature annealing at 923 K for 2 h was performed to further evaluate the structural stability of the epitaxial layers.

Benefiting from low-temperature growth, as shown in Fig. 3b, no obvious defects are observed in the transmission electron microscopy (TEM) image of the epitaxial cross-section. Figure 3c, d present the secondary ion mass spectrometry (SIMS) and atomic force microscopy results, from which it can be seen that the elemental compositions were well-controlled during epitaxial growth. However, the epitaxial surface reveals a distinct 3D island-like growth mode with a roughness of approximately 0.71 nm, rather than the ideal 2D growth mode. After annealing, significant crystal degradation is observed (Fig. 3e, f): the photoluminescence (PL) peak exhibits a blue shift of approximately 30 nm, with its intensity reduced by around 58%. The full width at half maximum (FWHM) also broadens by about 4 nm, and the indium atom concentration decreases from 35.6 to 33.0%. These data represent statistically averaged results calculated across the entire 6-inch wafer. When estimating the composition via measured photoluminescence spectra, the composition is derived based on the band gap width corresponding to the emission wavelength. Although this method introduces deviations from the actual quantum gain, it still allows for the observation of relative compositional changes.

Temperature directly affects atom behavior, and annealing can provide sufficient activation energy for indium segregation, desorption, and indium-gallium intermixing^44^. To visualize the correlation between interface structures and indium distribution, atomic-scale characterization of this sample was performed using a spherical aberration-corrected transmission electron microscope (Spectra 300), as presented in Fig. 5a–d. High-angle annular dark-field (HAADF) imaging—capable of resolving indium atom distribution—reveals distinct segregation: indium atoms are unevenly distributed within InGaAs quantum wells, diffusing outward from the center, with central content significantly lower than at the boundaries. Thus, it is evident that although the low-temperature-grown Sample 1 can achieve a 1.2 μm gain, its crystal structure exhibits relatively poor crystal quality and thermal stability. This directly reduces the effective indium composition post-annealing (manifested as a photoluminescence blue shift) and increases non-radiative recombination centers, leading to decreased PL intensity. Additionally, defects and impurities modify the quantum well band structure, introducing additional energy levels and broadening the FWHM.

**Fig. 4:**
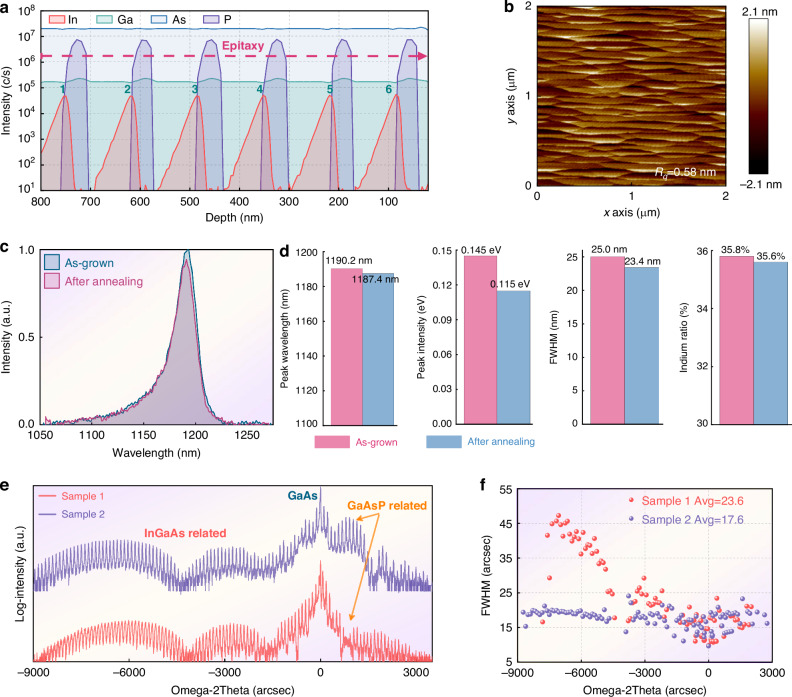
Epitaxial growth and characterization of Sample 2. **a** Measured secondary ion mass spectrometry (SIMS); **b** Atomic force microscope; **c** Photoluminescence spectrum before and after annealing; **d** Statistical PL peak wavelength, peak intensity, full width, and indium component at a six-inch wafer; **e** High-resolution X-ray diffraction of the two samples; **f** Statistical full width at half maximum of different diffraction peaks

**Fig. 5:**
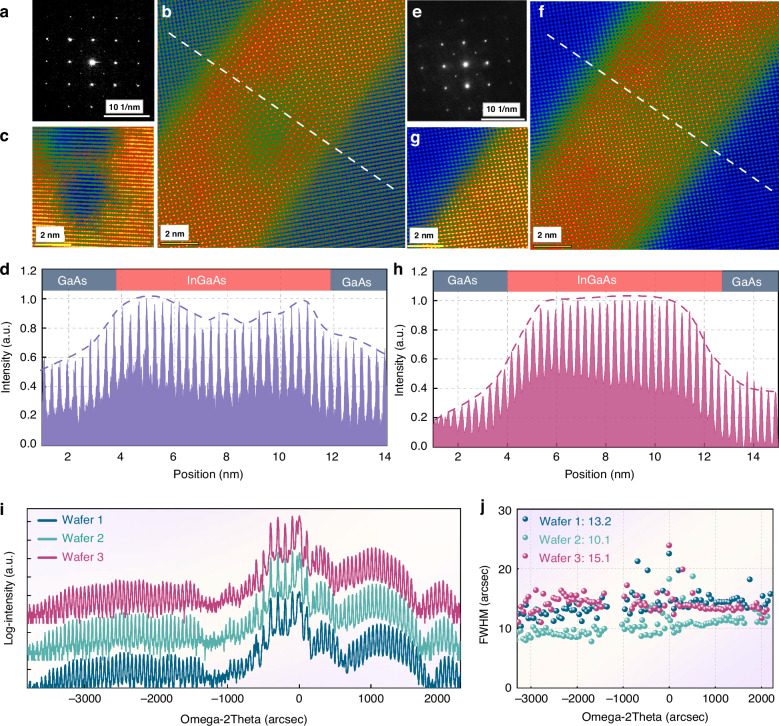
Atomic-level microscopic analysis of the two samples. Sample1. **a** Electron Diffraction Pattern; **b**, **c** HAADF image; **d** The atomic intensity distribution on the cross-section of the quantum well; Sample2: **e** Electron Diffraction Pattern; **f**, **g** HAADF image; **h** The atomic intensity distribution on the cross-section of the quantum well; **i**, **j** XRD diffraction and their peak position FWHM of three full-structure wafers from the same furnace batch

To address this issue, the potential of variable-temperature growth is further explored. During the growth of the active region, the growth temperature of the InGaAs layer remains fixed at 803 K, while that of the GaAsP layer is increased to 923 K. The temperature adjustment is performed within the GaAs insertion layer. The metalorganic sources remain unchanged. As observed in Fig. 4a, b, Sample 2 grown under variable temperatures exhibits an ideal 2D step-flow growth mode, with its roughness reduced to approximately 0.58 nm. The photoluminescence after annealing also exhibits better thermal stability and optical gain capability (Fig. 4c, d): the average PL wavelength shows only a slight blue shift of approximately 3 nm, changing from 1190 nm to 1187 nm. Rather than broadening, the FWHM decreases by 0.16 nm, and the calculated indium concentration decreases by only 0.2%. Figure 5e–h show the HAADF images, where a uniform distribution of indium atoms within the quantum wells can be observed, demonstrating that the indium segregation issue is significantly suppressed.

High-resolution X-ray diffraction (HRXRD) was further used to characterize the crystal quality of the two samples, as shown in Fig. 4e, f. It can be observed that Sample 2 exhibits a narrower FWHM, indicating that its internal interface roughness is significantly lower than that of Sample 1^47^. The XRD results were fitted using Bruker^TM^ RADs software, and the fitted phosphorus composition in the GaAsP layer of Sample 2 is 11%, compared to approximately 0.22% for Sample 1. By examining the GaAsP-related diffraction peaks, it is evident that the peak intensity of Sample 2 is significantly stronger, confirming that a higher phosphorus content is incorporated under high-temperature growth conditions. For the GaAsP layer, tensile strain will lead to an increase in its actual lattice constant, and the composition calculated via XRD is smaller than its true value. The above results indicate that PH₃ fails to dissociate sufficiently in Sample 1, leading to insufficient phosphorus incorporation and inadequate strain compensation. For comparison, Sample 2 adopts a variable-temperature growth method: it utilizes low-temperature growth to suppress indium atom migration, and the QWs undergo multiple annealing processes during repeated temperature ramping cycles, which reduces crystal defects. Employing a higher temperature for growing the GaAsP layer promotes phosphorus incorporation.

Essentially, effective strain compensation is the key to improving the crystalline quality and thermal stability. When an InGaAs layer is grown on a GaAs substrate, its larger lattice constant induces strong in-plane compressive strain in atomic bonds, shortening bond lengths below their intrinsic values and accumulating significant elastic strain energy. This energy is the primary driver of misfit dislocation formation and structural relaxation. Conversely, the subsequently deposited GaAsP layer—with a smaller lattice constant—undergoes in-plane bond stretching. The alternating arrangement of compressive strain and tensile strain achieves a near-zero average bond length and net strain energy. This atomic-scale energy balance strongly suppresses dislocation nucleation and propagation by acting as alternating energy barriers that hinder dislocation movement.

### Chip and second harmonic performance

**Fig. 6:**
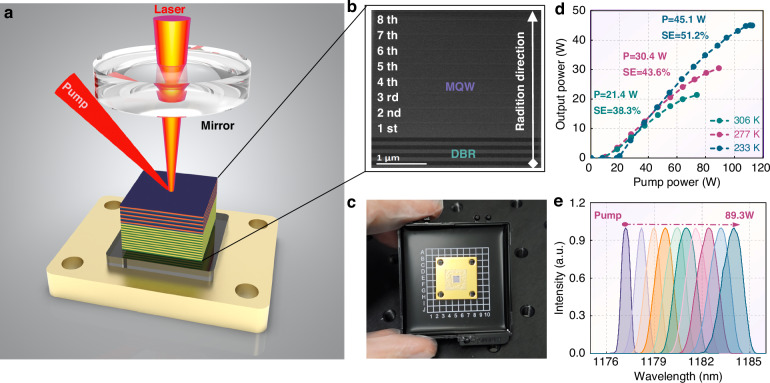
High power 1180 nm CW performance. **a** The adopted package structure and resonator for 1.2 μm operation. **b** TEM images of test chip, with 8-period InGaAs quantum well; **c** A kind of packaged VECSEL chip; **d** Output power versus pump, measured by (Coherent^TM^, PM150-50C) power meter. **e** Spectrum versus pump, measured by Yokogawa^TM^ AQ6370D

Subsequently, full-structure growth was carried out for the designed gain chip, with three wafers grown simultaneously using the variable-temperature growth method. The MOCVD equipment employed is capable of simultaneously growing 8 $\times$ 6-inch or 15 $\times$ 4-inch wafers, enabling rapid mass production. As shown in Fig. 5i, j, the XRD patterns of the three full-structure wafers exhibit similar diffraction peak positions and sharp diffraction peaks, with FWHMs all around 10–15 arcsec, confirming that they all possess good crystal quality. To evaluate the chip’s performance, a semiconductor-diamond packaging process was developed for its encapsulation, with the structure depicted in Fig. 6a. The detailed process is provided in ref. ^37^. After packaging, wet etching was employed to remove the substrate, as shown in Fig. 6b, c, leaving only the active region with a thickness of several micrometers.

**Fig. 7:**
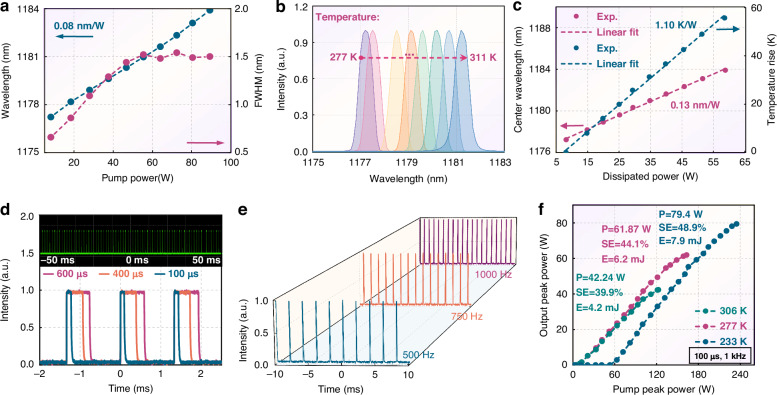
High energy 1180 nm QCW performance. **a** Central wavelength and FWHM versus pump; **b** Spectrum versus cooling temperature; **c** Wavelength and increased temperature versus dissipated pump powers. **d**, **e** Measured oscilloscope train with a Rohde & Schwarz™ oscilloscope with a bandwidth of 200 MHz; **f** Emission peak powers under different temperatures

The VECSEL performance is first evaluated under continuous-wave (CW) operation. As shown in Fig. 6d, a power of 30.4 W and a slope efficiency (SE) of 43.6% can be achieved at a coolant temperature of 277 K. When the temperature is lowered to 233 K, the CW power and slope efficiency further increase to 45.1 W and 51.2%, respectively. Lower temperatures enhance the wavelength offset between the quantum well gain and the longitudinal confinement factor, leading to an increase in the threshold pump power to ~19 W. At the maximum output power, the wall-plug efficiency of the laser is around 16%. To further enhance efficiency, a promising strategy is to adopt in-well pumping, which may help mitigate the limitations arising from the quantum defect and the pump source. Additionally, as the pump power increases from the threshold to 89.3 W, the central spectrum shifts from 1177.2 nm to 1183.9 nm, with an approximate redshift rate of 0.08 nm/W (Figs. 6e and 7a). At low pump powers, the spectral width gradually broadens from 0.67 nm to 1.5 nm as the carrier density increases. The gain will be saturated as pump power increases further. Additionally, most cavity modes that meet the threshold condition are activated at high pump, and the mode competition reaches a dynamic equilibrium, and the spectral width is stabilized.

Employing the spectral drift method, we further evaluated the thermal resistance, with Fig. 7b, c showing the spectrum as a function of temperature. When the temperature increases from 277 K to 311 K, the central spectrum shifts from 1177 nm to 1181.2 nm, with a shift rate (δλ/δT) of approximately 0.12 nm/K. Based on the temperature-dependent shift of the central wavelength and the lasing spectrum shift with dissipated power, we extracted the temperature increment using the formula *T* − *T*_*0*_ = (δ*λ*/δ*T*)/Δ*λ*, where the dissipated power is defined as the difference between the absorbed pump power and the output power. The thermal resistance, obtained by extracting the slope, is found to be only 1.1 K/W.

Apart from generating yellow SHG, the 1.2 μm laser is also extremely sought after in C–H bond photoacoustic tomography^48^, remote sensing, and space communication^49^. The high-energy emissions, which possess substantial attractiveness and potential utility within the realm of these applications, impel us to further evaluate the quasi-continuous wave (QCW) performance. As graphically illustrated in Fig. 7d–f, the VECSEL exhibits the capacity to function with tunable repetition frequencies and duty cycle parameters by manipulating the pump strategy. When the pulse width and repetition rate are set to approximately 100 μs and 1 kHz, a peak power of 79.4 W and a single-pulse energy of 7.9 mJ can be achieved. Similar to the CW operation, the threshold pump peak power increases significantly with decreasing temperature. For comparison, a full-structure chip was also fabricated using the identical growth protocol as Sample 1, as detailed in Fig. S2 in the Supporting Information. The chip exhibits an extremely low slope efficiency (~7%) and a CW power of only ~2.9 W. Tables S1 and S2 present a summary and compare the key material and device parameters for the growth methods. It can be observed that the variable-temperature growth method affords superior crystal quality and fewer defects in the fabrication of high-strain materials, thereby enabling a substantial enhancement in chip performance.

**Fig. 8:**
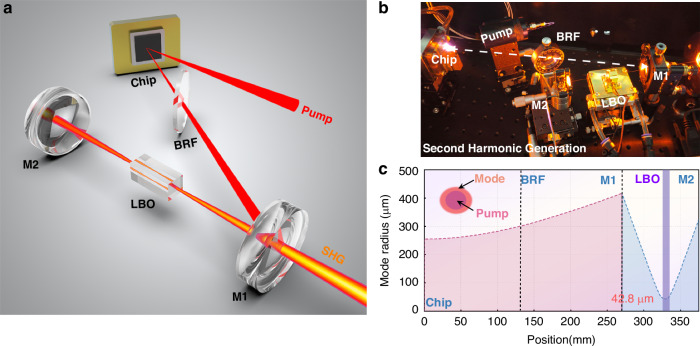
V-shaped resonator for yellow-SHG. **a**, **b** The designed and built V-shaped resonator for yellow-SHG; **c** Calculated fundamental mode radius within the cavity

**Fig. 9:**
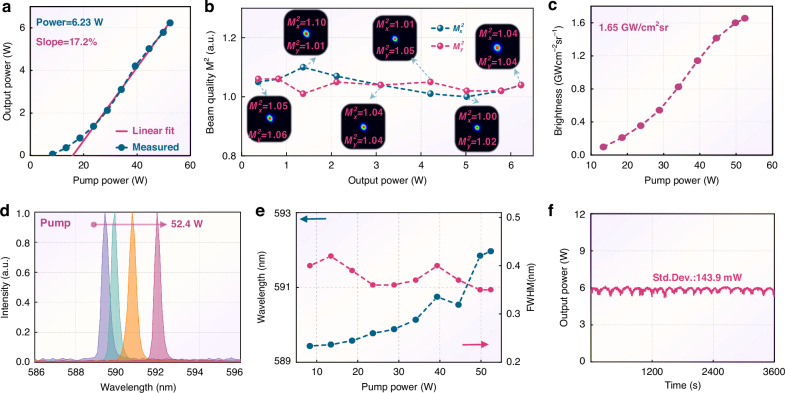
High brightness yellow-SHG performance. **a** SHG power versus pump, measured by (Ophir^TM^, 12A-V1) power meter; **b** Far-field pattern, and beam quality at various SHG power positions, proving the VECSEL operates in the near-diffraction-limited fundamental mode; **c** The calculated brightness; **d**, **e** SHG wavelength and FWHM versus pump, measured by Ocean Optics^TM^ spectrometer; **f** Monitored power fluctuations for 1 h

Furthermore, a V-shaped resonant cavity is constructed to test the SHG potential of the developed chip, and the physical image and the calculated mode radius within the cavity are shown in Fig. 8a–c. To facilitate single-mode operation, the pump spot radius is reduced to 340 μm, significantly smaller than the calculated fundamental mode radius at the chip (approximately 500 μm). With this configuration, higher-order transverse modes fail to achieve effective gain and are thus suppressed from oscillation, which enhances beam quality^50^. It can be observed in Fig. 9a that the maximum CW power exceeds 6.2 W, with a slope efficiency and a wall-plug efficiency reaching 17.2% and 5%. A beam-quality analyzer (Thorlabs™, BC207VIS/M) was used to comprehensively characterize the *M*² factor across different output power levels. As shown in Fig. 9b, the VECSEL operates in the fundamental mode near the diffraction limit, with an *M*² factor below 1.1. Brightness—a figure of merit encapsulating both output power and beam quality, and a maximum SHG brightness exceeding 1.65 GW cm^−2^ sr^−1^ was achieved (Fig. 9c), calculated using the formula $B=P/{\lambda }^{2}{M}_{x}^{2}{M}_{y}^{2}$.

As the pump power increases, the SHG central wavelengths redshift from 589.4 nm to 592 nm (Fig. 9d, e). The introduction of the birefringent filter (BRF) has compressed the linewidth, and the full-width at half-maximum (FWHM) of the spectra remains at approximately 0.4 nm, near the resolution limit of the spectrometer. For sodium atomic cooling and sodium beacon technologies, a spectral linewidth below the megahertz (MHz) level is typically required. To further narrow and stabilize the spectrum, the cascaded configuration of a BRF and a Fabry-Perot etalon has been verified to reduce the linewidth to the hundreds of kilohertz (kHz) range^51^. Further integration with Pound-Drever-Hall frequency stabilization technology enables wide-range frequency locking and mode-hop-free tuning. When the SHG power was set to approximately 6 W, its power stability was further evaluated, revealing a standard deviation of 144 mW over 1 h (Fig. 9f). These fluctuations potentially arise from reduced phase-matching efficiency caused by temperature variations and vibrations. To address these issues, a high-precision TEC controller, a customized enclosure, and an automatic control algorithm can be employed. With these measures, the stability of SHG is expected to be further enhanced to meet application requirements.

## Discussion

To transition VECSELs from laboratory to commercial use, factors including scalability, cost, throughput, and compatibility with industrial fabrication processes must be considered. A VECSEL typically comprises approximately 10 periods of quantum wells and over 30 pairs of λ/4 distributed Bragg reflector layers, with a total thickness of several micrometers. Growing such thick structures via MBE is extremely time-consuming, whereas MOCVD enables efficient fabrication through multi-wafer batch processing and delivers a far higher return on investment per wafer than MBE. The MOCVD growth of high-strain InGaAs/GaAs quantum wells has long been a key research focus in the fields of semiconductor epitaxy and laser technology, with numerous valuable studies dedicated to avoiding indium segregation and enhancing their crystal quality^33,36,45,52–54^. The use of strain-compensating layers has been theoretically and experimentally validated as effective for growing high-strain InGaAs materials. However, in MOCVD-based vapor-phase epitaxial growth, residual reactants are difficult to expel from the reaction chamber immediately, which can cause intermixing of indium and phosphorus atoms, leading to interface roughness. Additionally, low-temperature growth is required to suppress indium segregation, but the growth of GaAsP exhibits a nonlinear relationship between gas-phase and solid-phase proportions. Thus, precisely balancing the growth conditions for these two aspects is of critical importance.

Here, a GaAs insertion layer was employed to suppress the intermixing of indium and phosphorus atoms, and two distinct growth strategies were explored, and the potential of variable-temperature integrated growth is investigated. Utilizing low-temperature growth to suppress indium segregation, and employs a higher temperature for growing the GaAsP layer to promote phosphorus incorporation. Meanwhile, the quantum well undergoes multiple annealing processes during repeated temperature ramping cycles, which reduces crystal defects. The outcomes indicate a substantial improvement in the crystal quality and thermal stability. Subsequently, the test results show that the grown gain chips can attain CW power over 45 W and a slope efficiency greater than 50%. By further integrating a nonlinear crystal into the resonator, a yellow SHG with a CW power of approximately 6.2 W and nearly diffraction-limited beam quality (*M*² < 1.1) is achieved. The calculated brightness reaches approximately 1.65 GW cm^−2^ sr^−1^, which is comparable to or even surpasses some solid-state or fiber lasers. By introducing intracavity pulse modulation, higher peak power may be generated, which in turn further enhances the SHG efficiency and power. This paper demonstrates the effectiveness of MOCVD in batch-growing 1.2 μm high-strain VECSEL gain chips and verifies its potential for achieving high-brightness yellow sources.

**Fig. 10:**
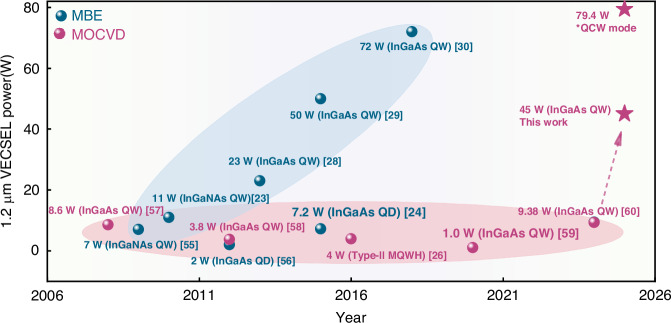
The leading developments concerning the 1.2 μm VECSELs. Blue and purple symbols denote the gain chips fabricated based on MBE and MOCVD, respectively

Figure 10 compares leading 1.2 μm high-power VECSELs^23,24,26,28–31,55–60^, although the data presented here mark a significant advancement for MOCVD-based 1.2 μm VECSELs, a performance gap persists compared to MBE-grown counterparts. To bridge this gap and match MBE’s quality, several avenues warrant further exploration. First, further optimizing the growth temperature of active quantum wells is crucial to “freeze-in” strain and suppress defect nucleation. Second, implementing advanced strain-engineering techniques—such as inserting strain buffer layers or utilizing patterned substrates—can effectively block and deflect threading dislocations away from the active region. Finally, enhancing in-situ monitoring and control for precise growth front management, combined with reactor design optimizations to minimize gas-phase memory effects, will be essential to achieving the required interface quality and compositional uniformity.

## Materials and methods

### Chip thermal management and pump source

The heat sink is in direct contact with the liquid cooling channel for heat dissipation. A S&A™ CW-5200 water chiller is initially used to cool the chip above 273 K. Subsequently, to achieve lower temperatures, pentafluoropropane coolant is employed, regulated by an electrically driven control system (LNEYA™, Sundi-1075WY). These two cooling configurations exhibit temperature control accuracies of ±0.3 °C and ±0.5 °C, respectively. A fiber-coupled laser diode module operating at 806 nm (FWHM ~ 1.2 nm) is used as the pump, delivering a maximum pump power of 198 W via a 200 μm fiber. The electro-optical efficiency and slope efficiency are approximately 40% and 22 W/A, respectively.

### V-shaped resonator for yellow-SHG

The two arm lengths of the V-shaped resonator are about 270 mm and 110 mm, with an included angle of about 10 °. The folding mirror M1 has been fabricated with an 1180 nm-high reflectivity and 590 nm-high transmittance film coating. The rear mirror *M*^2^ exhibits high reflectivity at both 1180 nm and 590 nm. A BRF is inserted into the long arm to narrow the linewidth and adjust the polarization state, thereby optimizing SHG efficiency. A type-I phase-matched lithium triborate (LiB₃O₅, LBO) crystal is employed to generate the second harmonic. Given that SHG efficiency is dependent on the fundamental frequency power density, the LBO crystal is positioned at the beam waist, where the calculated spot radius is ~42.8 μm.

## Supplementary information

Supplemental Material for Over 1.65 GW cm^−2^ sr^−1^ Brightness 590 nm Yellow Second-Harmonic Generation in MOCVD-Grown High-Strain InGaAs/GaAs Quantum Well VECSEL

## Data Availability

Data underlying the results presented in this paper are not publicly available at this time but may be obtained from the authors upon reasonable request.
