# Supplementary material for: Over 1.65 GW cm−2 sr−1 brightness 590 nm yellow second-harmonic generation in MOCVD-grown high-strain InGaAs/GaAs quantum well VECSEL
Source: Light Sci Appl. 2026 Mar 10;15:161. doi: 10.1038/s41377-026-02230-8 (PMC12976354; doi:10.1038/s41377-026-02230-8)
Supplement: Supplementary file 1 — Supplemental Material for Over 1.65 GW cm−2 sr−1 Brightness 590 nm Yellow Second-Harmonic Generation in MOCVD-Grown High-Strain InGaAs/GaAs Quantum Well VECSEL [file 41377_2026_2230_MOESM1_ESM.pdf]

# Supplementary Information for:

# Over 1.65 $GWcm^{-2}sr^{-1}$ Brightness 590 nm Yellow Second-Harmonic Generation in MOCVD-Grown High-Strain InGaAs/GaAs Quantum Well VECSEL

***Zhicheng Zhang[1*], Wenbo Zhan[2], Yao Xiao[2], Chen Luo[3], Hao Zhou[3], Wenfan Yang[2], Yang Cheng[1], Hao Yu[1,2], Quanling Li[2], Xiao Li[1], Chaofan Zhang[1*] and Jun Wang[1,2,3*]***

[1]*College of Advanced Interdisciplinary Studies, National University of Defense Technology, Changsha 410073, China*

[2]*Suzhou Everbright Photonics Co., Ltd, Suzhou 215163, China*

[3]*College of Electronics and Information Engineering, Sichuan University, Chengdu 610064, China*

**Contents:**

1. **The energy band diagram of the designed chip.**

2. **Performance comparison between two growth methods.**

## 1. The energy band diagram of the designed chip

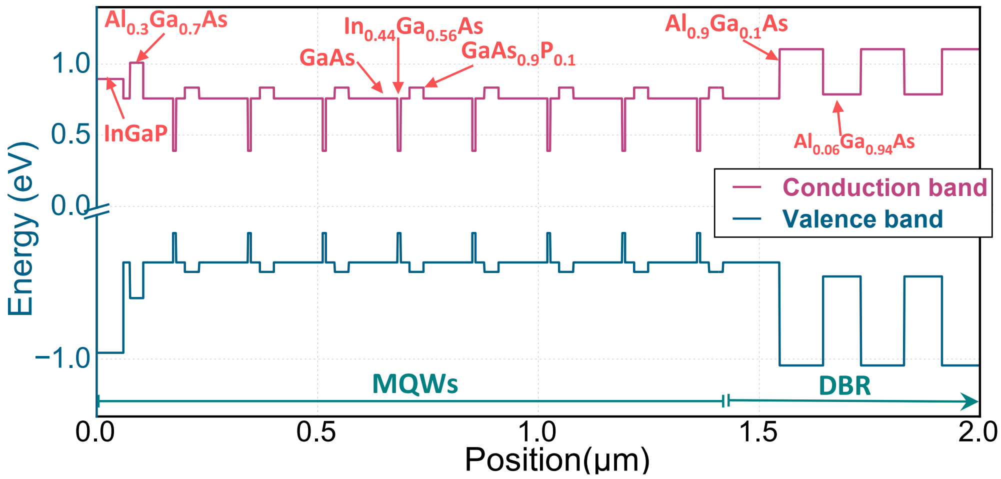


**Figure S1| The calculated energy band structure of the design chip.**

## 2. Performance comparison between two growth methods

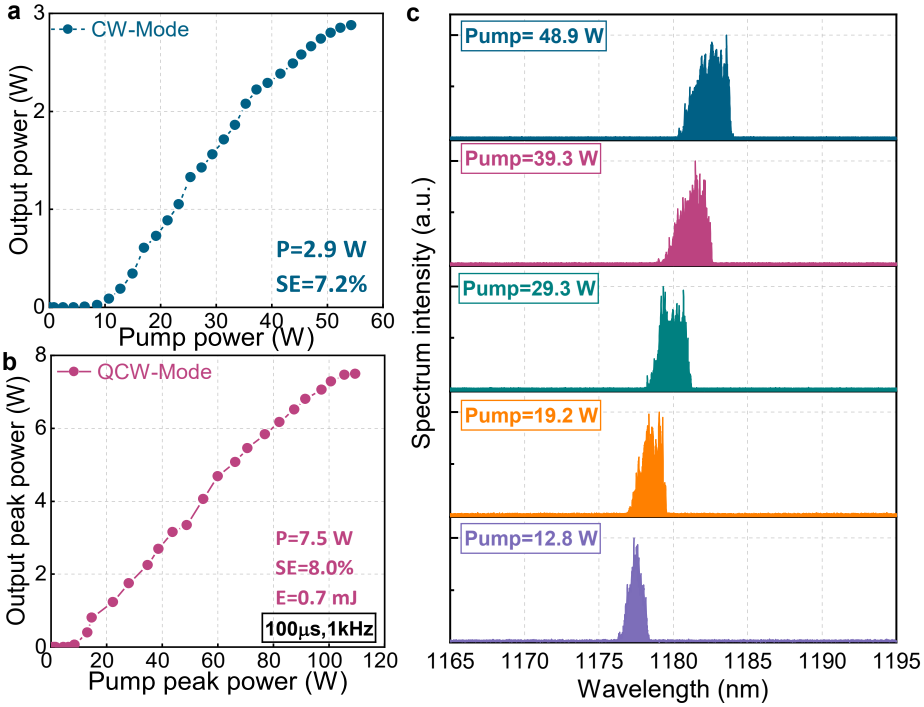


**Figure S2| Chip Performance: (a) Continuous-wave (CW) output power; (b) Quasi-continuous-wave (QCW) output peak power; (c) Spectral variation with pump power.**

To facilitate comparison with the chip performance, a full-structure chip was fabricated using the identical growth protocol as Sample 1. As observed in **Figure S2**, the chip exhibits a

**Table S1| Comparison of key material parameters between the two samples.**

| Material parameters | | low-temperature | variable-temperature |
|---|---|---|---|
| As-grown | PL wavelength | 1187.6 nm | 1190.2 nm |
| | PL intensity | 0.142 eV | 0.145 eV |
| | PL FWHM | 23.6 nm | 25.0 nm |
| | XRD FWHM | 23.6 arcsec | 17.6 arcsec |
| After annealing | PL wavelength | 1157.4 nm | 1187.4 nm |
| | PL intensity | 0.06 eV | 0.115 eV |
| | PL FWHM | 27.7 nm | 23.4 nm |

**Table S2| Comparison of chip performance between two growth methods.**

| Chip performance | | low-temperature | variable-temperature |
|---|---|---|---|
| CW mode | Power | 2.9 W | 30.4 W |
| | Threshold | 10.8 W | 8 W |
| | Slope efficiency | 7.2% | 43.6% |
| QCW mode | Peak Power | 7.5 W | 61.9 W |
| | Threshold | 8.5 W | 6.0 W |
| | Slope efficiency | 8% | 44.1% |

continuous-wave (CW) output power of only ~2.9 W and a peak power of 7.5 W—performance metrics significantly inferior. This discrepancy is attributed to the abundance of defects in the epitaxial structure, which deplete carriers and induce non-radiative recombination. This mechanism is validated by the extremely low slope efficiency (~7%) and a moderate increase in threshold pump power. **Tables S1** and **S2** present a summary and compare the key material and device parameters for the two samples. It can be observed that the device performance and material characterization results have good consistency. The variable-temperature growth method affords superior crystal quality and fewer defects in the fabrication of high-strain materials, thereby enabling a substantial enhancement in chip performance.
